# Unveiling Potassium and Sodium Ion Dynamics in Living Plants with an *In-Planta* Potentiometric Microneedle Sensor

**DOI:** 10.1021/acssensors.4c01352

**Published:** 2024-09-18

**Authors:** Qianyu Wang, Águeda Molinero-Fernández, José-Ramón Acosta-Motos, Gastón A. Crespo, María Cuartero

**Affiliations:** † Department of Chemistry, KTH Royal Institute of Technology, Teknikringen 30, SE-114 28 Stockholm, Sweden; ‡ UCAM-SENS, Universidad Católica San Antonio de Murcia, UCAM HiTech, Avda. Andres Hernandez Ros 1, 30107 Murcia, Spain; § Plant Biotechnology for Food and Agriculture Group (BioVegA), Universidad Católica San Antonio de Murcia (UCAM), 30107 Murcia, Spain; ∥ Plant Biotechnology, Agriculture and Climate Resilience Group, Associate Unit of R&D+i CSIC-UCAM, 30100 Murcia, Spain

**Keywords:** wearable microneedle sensor, plant monitoring, ion detection, sap collection, basil potassium and sodium, salt stress

## Abstract

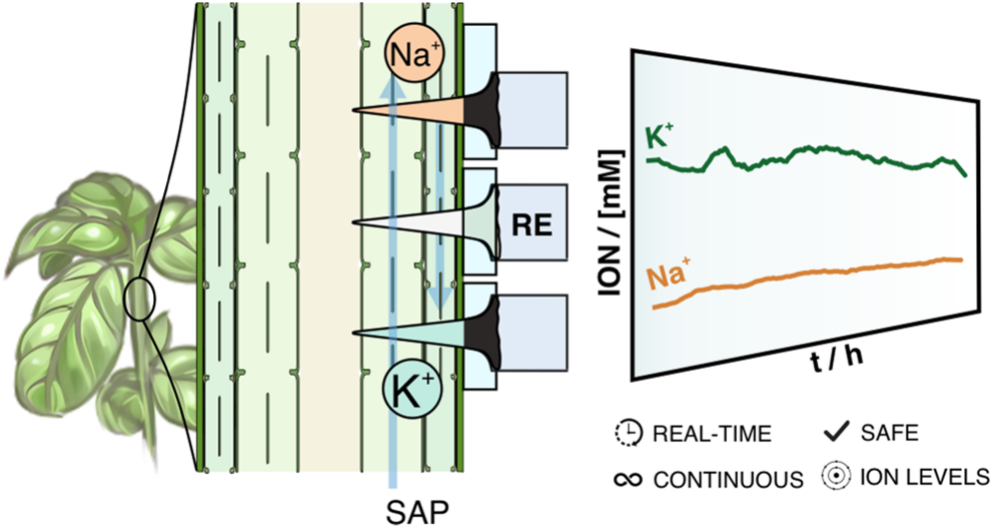

Potassium and sodium ions (K^+^ and Na^+^) play crucial roles in influencing plant growth and health status. Unfortunately, current strategies to determine the concentrations of such ions are destructive for the plants because it is necessary to collect/extract the sap for further analysis and produce either scattered or delayed results. Here, we introduce a new potentiometric dual microneedle sensor for nondestructive, real-time, and continuous monitoring of K^+^ and Na^+^ concentrations in living plants. The developed sensors show a response time <5 s, close-to-Nernstian slope (∼55 mV dec^–1^), resiliency to five insertions on the stem, good repeatability (max. %RSD = 0.3%) and reversibility (max. %RSD = 3%), appropriate continuous operation for 24 h, and linear range of responses that cover expected plant physiological levels (5–50 mM for Na^+^ and 50–120 mM for K^+^). Moreover, the accuracy was successfully investigated by comparing the results provided by the microneedle sensors to those obtained by a standard reference method (e.g., ion chromatography). Finally, we demonstrate that the developed analytical device is capable of tracking K^+^ and Na^+^ transportation from the hydroponic solution to the stem within 5–10 min. This research will contribute to establishing a new generation of analytical platforms for smart agriculture offering real-time information.

According to the Food and Agriculture Organization of the United Nations, it is essential to increase food production to feed the expanding global population (it is estimated that the world population will reach 9.7 billion by 2050).^1^ Agriculture innovation has been claimed to be essential for such a purpose to be materialized. Wearable plant sensors are emerging as new tools for crop production and management, among other matters.^2^ These sensors can monitor plant growth, diseases, and environmental pollution by determination of relevant chemical markers.^2^ Notably, ions are known to be crucial for optimum plant growth, development, and reproduction. For example, potassium ion (K^+^) is involved in many physiological activities, such as enzyme activation, protein synthesis, and photosynthesis, enhancing stomatal aperture.^3^ A well-known symptom from K^+^ deficiency is the yellowing between leaf tips and veins.^4^ Also, high levels of ions can injure plants. The sodium ion (Na^+^) has the most adverse effects on plant growth due to its detrimental influence on plant cell metabolism.^5^ Indeed, optimum K^+^/Na^+^ homeostasis is critical to maintain plant growth and yield by activating enzymatic reactions and keeping water balance. Accordingly, early detection of ion’s deficiency or concentration-related toxicity allows us to take the right actions that minimize stress on the plant and promote efficient growth.^6^

In vascular plants, ions are mainly present in tissues such as the xylem, where water and dissolved ions travel up to other parts of the plant (e.g., leaves). Then, in the phloem, essential ions are transported at low concentrations compared to other substances (e.g., sugars), and this movement can occur in different directions.^7^ Overall, the fluids within these plant tissues, commonly called sap, offer a valuable matrix for analyzing the plant’s biochemical constituents. Thus, there is a strong demand for an efficient sensing platform that can monitor ion fluctuations in sap directly on-site (i.e., *in-planta*) and without damaging the plant integrity. However, to the best of our knowledge, no sensor that covers these requirements has been developed yet. As a result, methods for sap chemical analysis are still focusing on assays performed in central laboratories after its extraction,^8^ being time-consuming, labor-intensive, and thus inadequate to acquire real-time and on-site data. Also, characterization of other sap parameters has been demonstrated through thermometric and heat pulse techniques, although these are indirect measurements, suffer from external influences, present limited spatial resolution, and are potentially disruptive for the plant, among other drawbacks.^9,10^

Microneedle (MN) technology may overcome these challenges and become a useful tool for in situ and real-time plant health monitoring in a similar way that has occurred for humans and animals via transdermal measurements.^11^ Effectively, MN sensors have proved their potential for ions’ concentrations monitoring under the skin by analyzing the dermal interstitial fluid,^12,13^ while its application for plant monitoring remains underexplored. Recently, an MN sensor has been designed for profiling total ion fluxes by measuring the electrical conductivity of the sap.^14^ While being the most evident attempt addressing ions’ detection, this sensor cannot differentiate between ionic species but provides the total content, limiting its ability to reveal a complete biochemical and physiological picture.

Herein, we present a new dual K^+^/Na^+^ MN-based sensing platform for ion monitoring in living plants. Individual MNs are modified to provide both K^+^ and Na^+^ profiling through their simultaneous detection in sap punching the plant with the MN sensing device. *In-planta* studies were performed in living basil (*Ocimum basilicum*) after deep in vitro characterization of both analytical and physical features of the MN sensors. Notably, basil plants were used due to their adaptability to diverse environmental conditions, being an ideal model to study the plant response to high salt stress.^15^ Specifically, the MN sensors were employed to monitor ion fluctuations in basil under (i) salt stress and (ii) darkness. Importantly, a validation protocol is proposed and implemented for *in-planta* outcomes observed here. The presented dual MN sensor has enormous potential in terms of providing new analytical tools for ion concentration monitoring in plants. It will also accelerate the development of new wearable sensors for the new era of smart agriculture.

## Experimental Section

### Fabrication of the K^+^/Na^+^ MN-Based Sensors

**Figure 1:**
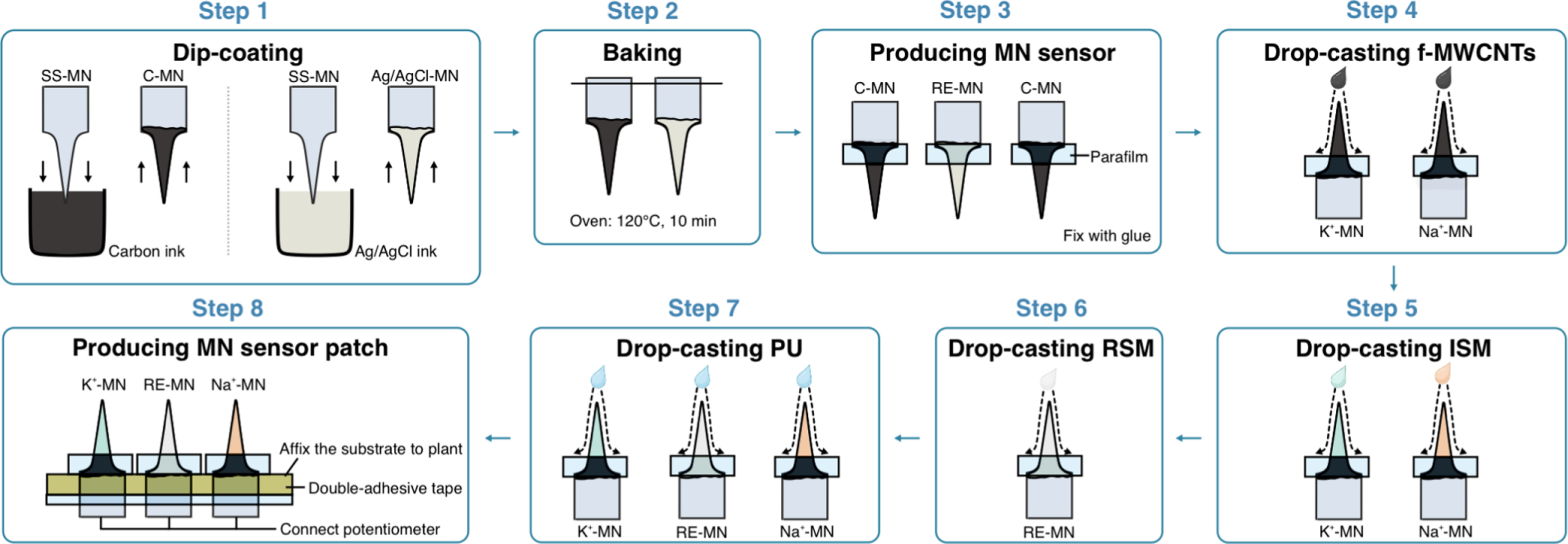
Schematics of the fabrication procedure of the patch based on the K^+^/Na^+^-MN sensors. SS = stainless steel. C = carbon. Ag/AgCl = silver/silver chloride. f-MWCNTs = functionalized multiwalled carbon nanotubes, ISM = ion-selective membrane, RSM = reference selective membrane, and PU = polyurethane.

The wearable patch consisted of a three MN-based electrode system (2 working electrodes for K^+^ and Na^+^, WE-MNs, together with a common reference electrode, RE-MN) assembled on a flexible parafilm substrate. The three MNs made of stainless steel were externally modified to create the electrodes, as shown in Figure 1. After each MN was cleaned with tetrahydrofuran (THF) to remove plastic residue, each one was dip-coated in the corresponding conductive ink (i.e., carbon [C] for the WE-MNs and silver/silver chloride [Ag/AgCl] for the RE-MN) and cured in an oven (120 °C, 10 min). After MNs were inserted into the parafilm substrate, they were glued by Loctite Super Glue (Henkel Norden AB) in the upper part, which will be used for the connections to the electronic reader. The bottom part was further functionalized to obtain the sensing MNs, as previously developed by our research group.^13^

For the WE-MNs, 10 layers of 4 μL of a solution of functionalized multiwalled carbon nanotubes (f-MWCNTs)^16^ in ethanol were drop-casted onto the C-covered MN. Drying steps of approximately 5 min were performed between each layer. Next, 3 layers of 4 μL of the corresponding ion-selective membrane (ISM) solution were drop-casted onto the MN. Drying steps were performed for 20 min after the first and second layers and 4 h after the final layer. The ISM compositions are detailed in the Supporting Information. For the RE-MN, 3 layers of 4 μL of the reference membrane cocktail were placed onto the Ag/AgCl-covered MN. Drying steps were also performed for 20 min after the first and second layers and 4 h after the final layer. Details on the membrane composition are provided in the Supporting Information. Finally, an external layer of 4 μL of polyurethane (PU) solution (33 mg in 1 mL of THF with 66 mg of bis(2-ethylhexyl) sebacate) was drop-cast to facilitate insertion and prevent the sensing components from being damaged by multiple insertions in the plant. Finally, the MNs were conditioned overnight in 0.01 M K^+^ (K^+^-MN), 0.01 M Na^+^ (Na^+^-MN), and 3 M KCl solution (RE-MN).

### In Vitro Characterization of the K^+^/Na^+^ MN-Based Sensors

Each type of WE (i.e., for K^+^ and Na^+^) was individually characterized against a RE-MN. Each WE-RE-MN pair was connected to the Lawson potentiometer via the metal crocodile clip in the edge of a Bayonet Neill-Concelman (BNC) coaxial cable. The electromotive force (EMF) of the electrochemical cell when immersing each pair in solutions of increasing ion (K^+^ or Na^+^) concentrations was measured to establish the corresponding calibration curve. Notably, for the confirmation of the appropriate RE-MN operation and the selectivity studies of the WE-MNs, a commercial double junction Ag/AgCl/3 M KCl reference electrode (6.0726.100, Metrohm AB, Sweden) was used as the RE. Experimental concentrations were converted into activities based on the two-parameter Debye–Hückel approximation for a formal inspection of the data.^17^

### Description of *In-Planta* Measurements with the K^+^/Na^+^ MN-Based Sensors

A portable, compact 8-channel potentiometer board equipped with wireless data transmission capability together with a customized mobile application was used to collect and process the *in-planta* data. These were previously designed and reported by our research group.^12^ In continuous observations, the smoothing of the raw signals was accomplished by using the Savitzky–Golay filter function in MATLAB. The basil plant (Organic, Himlajord) was purchased at a local supermarket. Most of the soil was removed (being sure that no roots were damaged), and the plant was immersed in a hydroponic solution in the laboratory. The ambient temperature was controlled to be 22.5 °C, and light–dark cycles of 12 h were implemented during the experimental period.

To prepare the hydroponic solution, 3 mL of commercially available hydroponic nutrition (Nelson Garden, Blomsterlandet, Sweden) was diluted in 1 L of tap water, according to the manufacturer’s instructions. Before *in-planta* measurements, a three-point calibration was performed for the patch containing the K^+^/Na^+^ MN-based sensors. The basil plants herein used were confirmed to be in intact conditions; i.e., the leaves were normally spread, the stems were healthy, and the roots were fully immersed in the hydroponic solution. Then, for the MN-based measurements, it is recommended to fix the basil by creating a plant support using a stick, approach the patch carefully, and insert the MNs carefully to reach the stem. Finally, the electrical connections (by crocodiles) were made, and the EMF was recorded. When the observations were finished, the MNs were smoothly pulled out and a postcalibration was accomplished. The pre- and postcalibrations were compared to confirm that there were no significant changes in the slopes and intercepts for K^+^ and Na^+^ quantification. These were constructed in terms of ion concentrations instead of activities to be able to access dynamic concentration profiles. Then, the stem sap was immediately extracted from the plant for further ion chromatography (IC) analysis.

### Plant Sap Collection and IC Analysis

A stem segment was cut from the plant. Tweezers were used to firmly grasp the exposed end of the stem and exert pressure to extrude the sap from both the xylem and phloem until a drop formed at its tip. The drop was quickly transferred into a microtube utilizing a micropipet (1–10 μL, Eppendorf Research Plus). The sample was stored in the freezer at −20 °C before analysis. For that, the collected sap was diluted using ultrapure water (Merck Millipore) in a centrifuge tube (1 mL) and employing a vortex mixer to adequately mix the solution for 5 min (speed level: 800 rpm). Since the collected sap may contain insoluble substances, such as plant tissue, centrifugation was required. Thus, the tube was centrifuged at 14,000 rpm for 5 min. Then, the supernatant was collected and diluted with water (900 μL in 11 mL of water), filtered (PTFE paper with 0.45 μm pores), and stored in the IC tube. IC measurements (50 Professional IC, Metrohm AB, Sweden) were carried out in triplicates. The eluent was 2.5 mM HNO_3_, the flow rate was 0.9 mL/min, the separation column Metrosep was C6–150/4.0, and the conductivity detector (2.850.9010, Metrohm) was used for cation quantification. Figure S1 summarizes the procedure for sap collection and validation in the IC.

### Preparation of Cross-Section Samples of Plant Stems for Microscopy Evaluation

Freehand sectioning was adopted to prepare the cross-section samples. First, a short segment of the stem was cut, which was ideally perpendicular to its long axis. The segment was firmly held, and a blade was used to carefully shave off thin sections. These cross-sections should be as thin as possible (approximately 60–100 μm). Then, the section was gently put onto a drop of water placed on the microscope glass slide, and the glass coverslip was slightly lowered onto it, avoiding trapping air bubbles. The sample was inspected in a microscope (Nikon Eclipse Ti2, Japan).

## Results and Discussion

**Figure 2:**
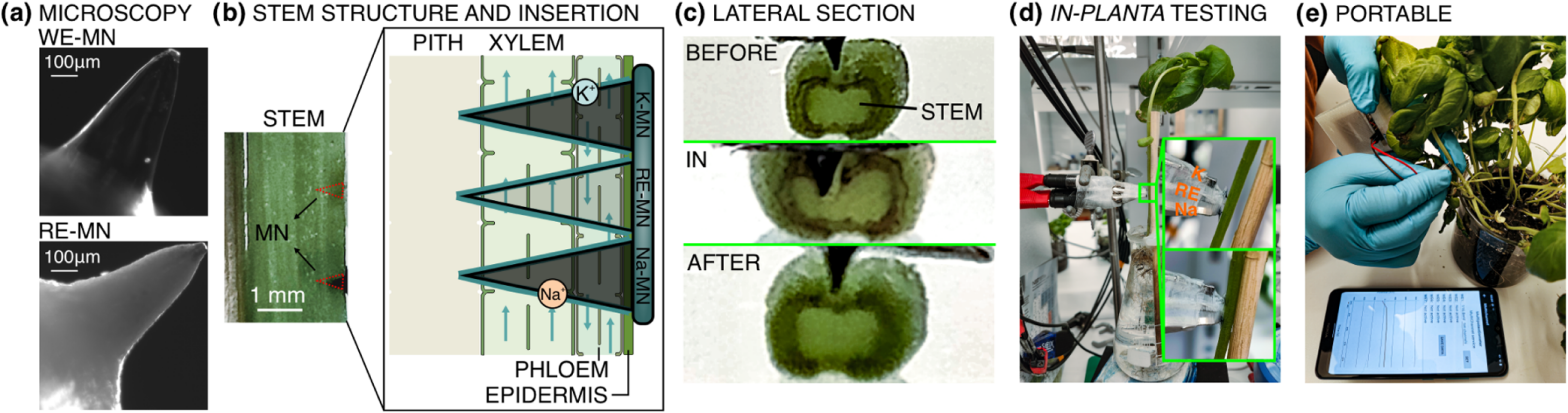
(a) Microscopic photos of WE-MN and RE-MN. (b) Diagram of the MN insertion into a basil stem and the schematic cross-section of the stem structure. (c) Images of the lateral section of the MN insertion procedure. (d) Experimental setup for *in-planta* testing. Inset: magnification of the MN approaching and insertion into the stem. (e) Real-time monitoring of K^+^ and Na^+^ concentration profiles using the MN sensor patch connected to a portable potentiometric board.

Real images of the patch are provided in Figure S2a. Briefly, both WE-MNs are conceived as potentiometric all-solid-state ion-selective electrodes composed of three layers: (i) a carbon layer that improves conductivity and attachment of the further layers to the MN; (ii) f-MWCNTs as the ion-to-electron transducer; and (iii) an ISM to provide selectivity to the target ion. The common RE-MN is also of the solid-state type and contains a Ag/AgCl layer covered by the PVB membrane. Both types of electrodes have an external PU layer that improves the potential stability and resiliency to plant insertion (see below). Figure S2b presents pictures of individual WE- and RE-MN. As observed, the PU layer entirely covers the MN and serves as a seal with the surrounding parafilm. The dimensions and geometry of the MNs were characterized by microscopy, as these are critical aspects to reach the sap while being minimally invasive and nondestructive for the plant. Images of WE-MN (K^+^-MN as an example) and RE-MN are shown in Figure 2a. Both MNs presented similar dimensions, with a height of 625 ± 5 μm, base width of 311 ± 8 μm, tip diameter of 30 ± 6 μm, and tip angle of 22° in the case of K^+^-MNs (*n* = 5), and a height of 627 ± 9 μm, base width of 301 ± 5 μm, tip diameter of 24 ± 2 μm, and tip angle of 22° in the case of RE-MNs (*n* = 5). According to previous studies, the revealed dimensions should allow effortless manual insertions in plant stems.^18^ However, because stem hardness is heterogeneous between different plants, the proper penetration of the MNs in the plant herein explored (i.e., basil) was deeply investigated.

Figure 2b shows two MNs (WE and RE) inserted into the stem together with an approximate tissue scheme considering the MN dimensions and the level of insertion from the real image. In principle, the MNs penetrated the epidermis, phloem, and xylem to reach the pith so that they could analyze the ion content in the sap as a mix of these three layers. Lateral images before, during, and after insertion (Figure 2c) confirmed that the MNs reached the pith. Interestingly, the penetration depth of the MNs, and therefore the tissue they reach/analyze can be conveniently adjusted for various plants by either changing the length of the MNs or the thickness of the substrate in where they are fixed. In Figure S3 in the Supporting Information, it can be observed that the penetration of the MNs generated a hole on the stem plane of approximately 88 μm × 219 μm. Such a small disruption did not impose adverse damage to the plant’s normal growth. Indeed, it is very common to have physical damage to plants due to their interaction with the natural environment, and scarcely plants can grow undisturbedly. As a result, plants have evolved the competence to close wounds/damages and activate tissue reparation, among other pathways.^19^

Figure 2d provides a picture of the experimental setup for *in-planta* real-time tracking of K^+^ and Na^+^ in basil stem. The basil plant was securely fastened to a wooden support using double adhesive tape. This prevents the movement of the plant during the measurement. The magnification of the picture shows how the MN patch is positioned in the desired area and then punched. Figure 2e illustrates the *in-planta* monitoring using a portable potentiometric board that sends the data via Bluetooth to a smartphone for visualization. The described setup was herein used for all of the *in-planta* measurements.

### In Vitro Characterization of the K^+^/Na^+^-MN Patch

The nature of the substrate and the need for an outer layer were investigated. The tested conditions are summarized in Table S1 (Supporting Information). The study evaluated not only the analytical performances of the MN sensors but also physical features crucial for further *in-planta* measurements, such as resiliency to plant insertion. Notably, this study was performed for the Na^+^-MN, and the results were extended to the K^+^-MN, as a similar performance was expected. As a first approach, a Na^+^ concentration range from 1 to 180 mM (log *a*_Na_ from −3 to −0.75) was used in these experiments, considering covering the expected Na^+^ levels in plants (20–30 mM).^20^

The nature of the substrate (silicone rubber or parafilm) was investigated by performing calibrations against a commercial double junction Ag/AgCl reference electrode in an ultrapure water background. The MN sensors (without the outer PU layer) set in the parafilm patch exhibited a higher slope than those in the silicone one (57.2 ± 2.1 versus 54.7 ± 1.0, Table S1). This could be connected to one of our findings when inspecting the patches using the microscope. In essence, a certain level of membrane detachment was found in the case of the silicone rubber substrate (Figure S4), which is often linked to the existence of a water layer at the transducer-membrane interface. Thus, the parafilm material was selected to prepare the MN sensor patch for subsequent experiments.

The performances of the WE-MN prepared with and without the external PU layer were investigated. The corresponding MN was calibrated against a commercial double junction Ag/AgCl reference electrode in an ultrapure water background. Similar slopes (57.2 ± 2.1 versus 57.4 ± 1.8 mV dec^–1^) and intercepts (260.0 ± 22.9 versus 304.7 ± 27.0 mV) were obtained in both cases (Table S1). Then, the calibrations were repeated after several insertions (up to 5) into the stem to study any effect of the presence of the PU external layer on the integrity of the sensor and, hence, the calibration graphs. Table S2 presents the observed calibration parameters and the percentages of variation with regard to the original calibration graph. As observed, minimum variations were obtained only in the case that the PU outer layer was present (0.5–11.2% without PU and 0.0–0.5% with PU).

**Figure 3:**
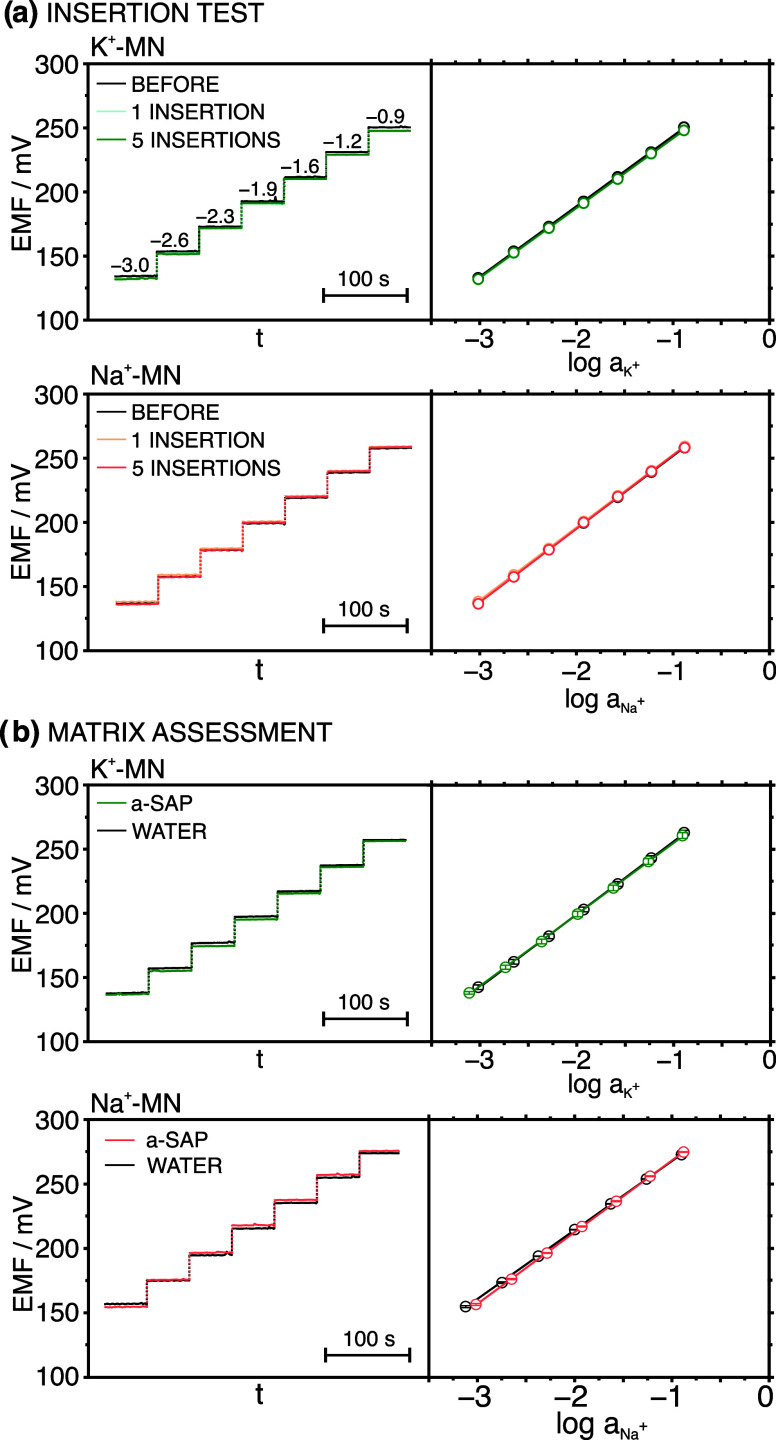
Dynamic potentiometric response and corresponding calibration curves of the K^+^/Na^+^-MN sensor (a) before, after 1, and 5 insertions into the plant, and (b) in artificial sap (a-SAP) and ultrapure water background.

By adopting the use of the outer PU layer for the preparation of all of the sensors (Na^+^-MN, K^+^-MN, and RE-MN) and with the patch made of the parafilm substrate, the resiliencies to the insertion of Na^+^ and K^+^ individual patches to five plant insertions were investigated. The observed dynamic responses and corresponding calibration graphs are presented in Figure 3a. Small relative standard deviations (RSD, %) were obtained for the calibration parameters: 0.5% and 0.4% for the slope and intercept of the K^+^-MN, 0.6% and 0.2% for the slope and intercept of the Na^+^-MN after five insertions. Indeed, when separate studies were performed with RE-MN, adequate results were also found. The initial potential provided by the RE-MN in 0.1 M KCl solution was recorded and then, the measurement was repeated after one and five insertions into the plant stem (Figure S5a, Supporting Information). Variations of 0.36 mV (first insertion) and 0.97 mV (fifth insertion) with respect to the original reading were observed. Moreover, no morphological changes (e.g., membrane detachment) were detected in the RE-MN. A microscopic image of the membrane–substrate boundary after the fifth insertion is provided in Figure S5b, revealing the absence of any detachment or adhered substance.

Subsequently, we confirmed the appropriate behavior of RE-MN by analyzing the calibration graphs of six patches containing Na^+^- and RE-MNs. In addition, the calibration for each patch was obtained utilizing a commercial Ag/AgCl reference electrode (RE-COM) instead of the RE-MN for comparison. The corresponding dynamic responses are shown in Figure S6, whereas the calibration parameters and response times are listed in Table S3. No significant differences were found between the slopes (maximum of 3% difference), linear range of responses (LRRs from 0.01 to 180 mM Na^+^ concentration, log*a*_Na_ from −5 to −0.75), limit of detections (LOD of 8.8 × 10^–6^ M for the RE-MN and 7.9 × 10^–6^ M for the RE-COM), and response times (3–5 s within the linear range of response). However, an offset in the range of 1.2–23 mV in the intercept was presented, which is associated with different constant potentials provided by each reference electrode. As a result, the developed RE-MNs were appropriate to be used in further measurements of Na^+^ and K^+^.

Repeatability tests were performed with individual patches for Na^+^ and K^+^. Three consecutive calibrations were conducted. The dynamic responses and corresponding calibration graphs are provided in Figure S7. The results showed negligible RSDs of 0.2% in the slopes and 0.1% in the intercepts for the K^+^-MN, and 0.2 and 0.1% for the Na^+^-MN. The reversibility was assessed by performing four calibration curves alternating increasing and decreasing concentrations of the target ion. The dynamic responses and the corresponding calibration graphs are provided in Figure S8. Reversible signals were observed for both ions, with a displacement of 1.1 and 0.3% for the slope and intercept for the K^+^-MN and 3.1 and 0.7% for the Na^+^-MN.

A selectivity study was conducted by considering the main ions present in the sap: Na^+^, K^+^, and Ca^2+^.^21^ According to previous studies, other inorganic and organic molecules (e.g., glucose) are not likely to influence potentiometric signals.^13^ Logarithmic selectivity coefficients were calculated for both K^+^- and Na^+^-MNs (*n* = 3) by the separate solution method.^22^ For all of the tested ions, selectivity coefficients were lower than the minimum value required for accurate measurements in sap (see Table S4). The results suggested that the MN sensors are, in principle, suitable for accurate measurements in sap. Nevertheless, to further confirm this, calibration graphs for Na^+^ and K^+^ were performed in artificial sap containing main and secondary ions at the following levels: 20 mM Na^+^, 120 mM K^+^, 20 mM Ca^2+^, 5 mM Mg^2+^, and 650 μM NH_4_^+^. While Na^+^, K^+^, and Ca^2+^ concentrations were equal to those observed by ion chromatography (IC) in the sap of our basil plant, Mg^2+^ and NH_4_^+^ concentrations were considered from the literature.^23,24^ The left panels in Figure 3b depict the dynamic responses observed for the first calibrations of Na^+^- and K^+^-MNs from a series of three consecutive experiments in both water and artificial sap backgrounds, whereas the right panels present the average calibration graphs. Table S5 in the Supporting Information collects the calibration parameters. No significant differences were observed in the slopes and intercepts provided in the two matrixes for both MN sensors.

Very low long-term drifts were obtained for the RE-MN as well as K^+^/Na^+^-MN individual patches (i.e., containing the RE-MN) when measuring in artificial sap for 24 h. Figure S9 in the Supporting Information presents the recorded potentials. Notably, in the case of the RE-MN, this was measured against the RE-COM. The RE-MN showed a drift of 0.009 mV h^–1^, while the K^+^ patch revealed a drift of 0.37 mV h^–1^ and the Na^+^ patch a drift of 0.15 mV h^–1^. These drifts are associated with changes in ion concentrations over time of 2.2 mM h^–1^ for the K^+^ patch (variation of 1.2%) and 0.16 mM h^–1^ for the Na^+^ patch (0.5%). While these variations are not expected to be physiologically relevant in the *in-planta* monitoring, a daily replacement or timely recalibration (estimated = every 12 h) of the patch is recommended to counteract the signal drift when pursuing the maximum accuracy in the long-term monitoring of the plant.

To fully characterize the LRR and LOD provided by the K^+^- and Na^+^-MN sensors, the concentration range used in the calibration graphs was extended from 10^–6.5^ to 10^–0.75^ M and the experiments were accomplished in both water and artificial sap. The dynamic recordings and corresponding calibration graphs for Na^+^ and K^+^ are displayed in Figure S10, with the analytical characteristics being collected in Table S6. When measuring in the same matrix with three consecutive calibrations, the variation was <0.2% in slopes and 1.5% in the intercepts for both the K^+^- and Na^+^-MNs. The study also showed a slight decrease in the LRRs and an increase in the LODs when moving from the ultrapure water background to artificial sap, indicating a certain matrix effect of the sap. Notably, while lower concentrations are expected in sap for Na^+^ than for K^+^ (20–30 versus 50–150 mM), in both cases, these are fully covered by the observed LRRs. Moreover, the Na^+^- and K^+^-MNs can be used at least two months after their preparation without a drastic effect on their performance. For example, for the K^+^-MN, all of the individual patches that were tested (*n* = 3) displayed Nernstian slopes >57 mV dec^–1^. To achieve such an excellent lifetime, the MNs were stored in a refrigerator and in air (i.e., the MNs were not immersed in a storage solution). The MNs were conditioned just prior to their usage.

### Analysis of Sap Samples

Na^+^ and K^+^ measurements in real sap samples were performed to investigate the accuracy of the developed MNs. Once more, individual patches for Na^+^ and K^+^ were used. Two sets of samples were analyzed: (i) extracted sap fortified with known Na^+^ and K^+^ concentrations (*n* = 2 for Na^+^ and *n* = 2 for K^+^) and (ii) raw extracted sap (*n* = 6). The extraction of the sap was performed by squeezing a segment of the basil’s stem (∼2 cm in length), as previously reported.^8^ For the fortified sap, the fluid was collected from multiple locations along the entire stem to obtain a sufficient volume (ca. 30 μL). Then, this pooled sap sample was fortified with a known concentration of the target ion (Na^+^ or K^+^), and recovery studies were performed. The main aim of these measurements was to confirm that added Na^+^ or K^+^ concentrations were recovered close to 100%, and therefore, no significant matrix effects existed in the MN-based measurements. Importantly, the sap samples were measured as collected without any pretreatment.

**Figure 4:**
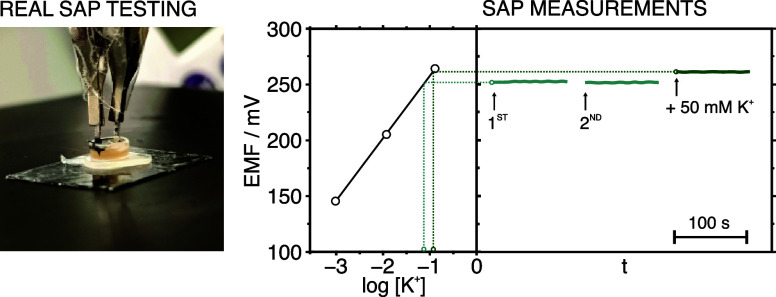
Experimental setup and procedure for K^+^ detection in the plant sap. This latter including a three-point precalibration curve, two consecutive measurements in the sample, and the potentiometric response after fortifying the sample with 50 mM K^+^ in the sap. The same protocol was followed for the Na^+^ measurements.

Figure 4 illustrates the steps performed for the analysis of known K^+^ levels added to sap samples. The same procedure was adopted for Na^+^. A real image of the experimental setup is also provided. The K^+^-MN patch was calibrated prior to the sap measurements following a three-point calibrant protocol (background: artificial sap). Then, the pooled sap sample was added into a microwell, and the patch was used to record the potentiometric signal twice for 100 s, dipping the patch in and out of the sap. Subsequently, the pooled sap was fortified with K^+^ (50 mM in the example shown in the figure) and the potential was again recorded for 100 s. The K^+^ values were calculated by extrapolating the average potential recorded for the latter into the calibration. The results are provided in Table S7 in the Supporting Information. Recoveries ranging between 102 and 110% were observed in all of the cases. These results demonstrated that sap matrix components did not significantly affect the potentiometric measurements, endorsing the trueness of the method and highlighting the potential of the methodology for *in-planta* usage. Moreover, a negligible 0.2% difference in the two initial potentials provided by the raw sap was obtained, validating the utilization of the MN for multiple recordings.

Next, plant sap samples were directly analyzed by the dual K^+^/Na^+^-MN patch, and the results were compared with those obtained by the IC as the reference method (Table 1). The K^+^/Na^+^ concentration of the sap was first quantified using the MN patch (recording the signal for 100 s), and then, the sample was transferred to the IC for analysis (*n* = 3). In all of the cases, the % of differences between the concentrations provided by both techniques were <12%. In addition, the paired sample *t*-test (*p* < 0.05) indicated that there was no statistically significant difference between the techniques for both K^+^ (*p* = 0.514, *n* = 6) and Na^+^ (*p* = 0.907, *n* = 6). The results indicated a high degree of agreement between the developed K^+^/Na^+^-MN patch and the reference method. Additionally, two MN sensors (e.g., K^+^-MN) showed a minimal discrepancy of 5% (mean diff.%) between them when measuring the same sap sample (Table S8).

**Table 1:** K^+^ and Na^+^ Concentrations in the Sap Collected from a Basil Plant Measured by MN-Based Sensors and IC

	K^+^ (mM)		
sap no.	MN^a^	IC^b^	difference (%)
#1	108.8 ± 0.2	105.2 ± 0.5	3.5
#2	108.7 ± 1.8	107.3 ± 0.2	1.2
#3	99.1 ± 1.4	94.3 ± 0.4	5.1
#4	103.2 ± 0.9	96.5 ± 0.2	6.9
#5	95.0 ± 0.5	90.5 ± 0.4	4.9
#6	121.2 ± 0.9	118.9 ± 0.2	1.9

	Na^+^ (mM)		
sap no.	MN^a^	IC^b^	difference (%)
#1	25.3 ± 0.4	24.4 ± 0.3	3.6
#2	26.0 ± 0.1	25.2 ± 0.1	3.0
#3	26.2 ± 0.3	25.9 ± 01	1.2
#4	26.6 ± 0.3	23.7 ± 0.1	12.2
#5	34.1 ± 0.1	35.1 ± 0.4	2.8
#6	21.5 ± 0.2	23.6 ± 3.6	8.9

a Average ± SD of a recording of 30 s.

b *n* = 3.

### *In-Planta* K^+^ and Na^+^ Monitoring with the Developed MN Patch

The suitability of the potentiometric dual MN sensing patch to provide *in-planta* K^+^ and Na^+^ concentration monitoring was assessed by using six basil plants. All of the basil plants were grown under similar conditions, except for plant #1, which was pretreated with 30 mM salt-stress conditions for 24 h. All of the plants presented similar morphological features, which are collected in Table S9 in the Supporting Information. To perform the *in-planta* measurements, the K^+^/Na^+^-MN patch was carefully inserted into the middle part of the corresponding stem, and stable potentiometric signals were recorded for 100 s. The potentiometric signals were then transformed into K^+^ and Na^+^ concentrations according to previous calibrations in artificial sap.

**Figure 5:**
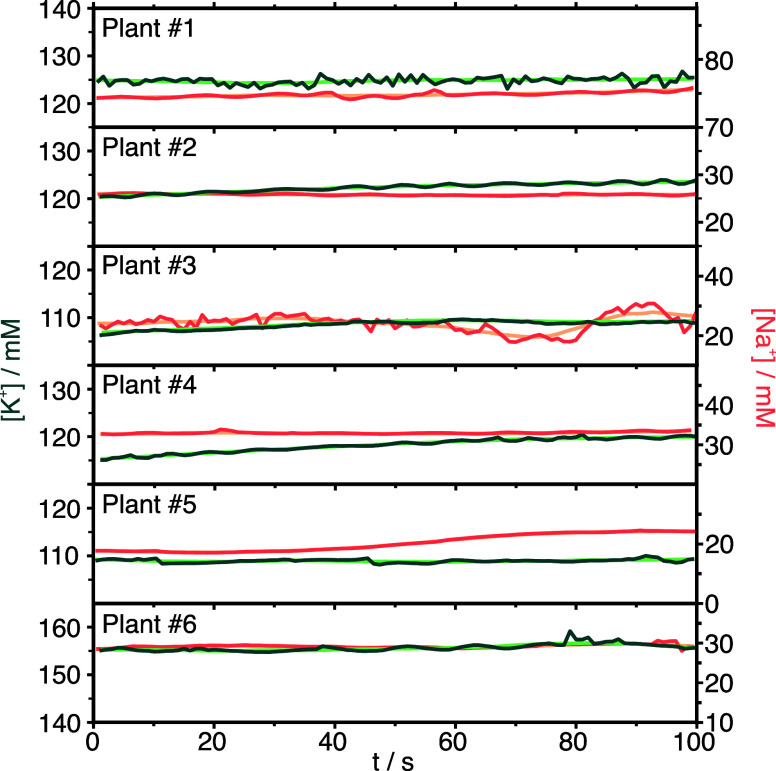
Dynamic profiles of K^+^ (green) and Na^+^ (orange) concentrations in six basil plants. Lines of light color indicate smoothing data.

The dynamic profiles for K^+^ and Na^+^ concentrations obtained for each plant are shown in Figure 5. After each of these measurements, the segment of the stem in which the patch was positioned was cut, and the sap was extracted using the squeezing method described in the Experimental Section. These samples were further analyzed by IC. All of the results are displayed in Table 2. Both techniques offered very similar values, with percentages of difference <15%, except for Na^+^ measurements in the basil plant #6. Notably, when measuring low Na^+^ concentrations, a small difference (e.g., a difference in concentration of 5 mM in plant #6) translates into a larger variation between both techniques (e.g., a percentage of variation of 21% in plant #6). A paired sample *t*-test revealed no significant differences between the values provided by both techniques at a confidence level of 95%: *p* = 0.545 (*n* = 6) with the calculated *t*_stat_ = 0.6 < *t*_critical_ = 2.2 for K^+^, and *p* = 0.445 (*n* = 6) with the calculated *t*_stat_ = 0.1 < *t*_critical_ = 2.2 for Na^+^. Overall, these results demonstrate the accuracy of the MN-based measurements in living plants.

**Table 2:** K^+^ and Na^+^ Concentrations Measured in Six Basil Plants by MN-Based Sensors and IC

	K^+^ (mM)		
plant no.	MN^a^	IC^b^	difference (%)
#1	125.06 ± 3.1	111.8 ± 0.4	11.9
#2	122.2 ± 1.5	113.4 ± 0.3	7.8
#3	108.08 ± 0.5	112.9 ± 0.8	4.2
#4	119.6 ± 0.9	104.4 ± 0.1	14.6
#5	109.3 ± 1.3	112.9 ± 0.2	3.2
#6	154.9 ± 1.6	148.4 ± 0.1	4.4

	Na^+^ (mM)		
plant no.	MN^a^	IC^b^	difference (%)
#1	76.6 ± 0.7	74.0 ± 0.4	3.5
#2	25.9 ± 0.2	24.5 ± 0.1	5.6
#3	24.3 ± 3.1	25.2 ± 0.1	3.4
#4	33.04 ± 0.3	32.3 ± 0.2	2.2
#5	24.3 ± 0.1	23.2 ± 0.2	4.9
#6	28.9 ± 0.3	23.9 ± 0.1	20.9

a Average ± SD of a recording of 30 s.

b *n* = 3.

Interestingly, when the concentration profiles were analyzed, low variabilities in the measured K^+^ and Na^+^ concentrations were noticed between basil plants that were grown under similar conditions. Effectively, basil plants #2–#5 presented ranges of K^+^ and Na^+^ concentrations from 104.4 to 122.2 and from 23.2 to 33.0 mM (considering both the MN sensors and IC), respectively. In contrast, a significantly higher concentration of Na^+^ was detected in basil plant #1 (76.6 mM), which was cultivated in a solution with a higher Na^+^ concentration than the rest. This result agrees with previous studies where basils were treated under salt stress.^25^

Any potential damage imposed by MN insertions into the plant during *in-planta* measurements was evaluated. In this regard, the MN patch was inserted for ca. 5 min into the central part of the stem, and then any postinsertion morphological features of the plant were inspected. First, it is noticeable how the plant did not show any negative effect as a reaction to the MN insertion even after 30 days (see Figure S11a in the Supporting Information). No changes in color, decrease in the number of leaves, or decrease in plant growth were observed. Just right after the disassembling of the MN from the plant, the created holes (referring to them as wounds from now on)^19^ were evident (Figure S11b). However, the wound closure was identified to start promptly (∼10 min) and continue in the following hours (Figure S11c). Moreover, in the subsequent days, gradual wound healing was observed (Figure S11d). This behavior agrees with previous studies.^26^ Interestingly, following a 30-day healing period, the plant wounds achieved complete closure, resulting in a residual hole of slightly larger diameter than the initial one. This is also aligned with previous findings.^27^

Regarding the plant subjected to salt stress (plant #1), from the third day of the treatment, it was detected that the leaves underwent a gradual shriveling, acquiring a yellowing color and being desiccated in around 1 week, similar as in other studies.^28^ Accordingly, we investigated if the developed MN sensing patch is suitable for monitoring ions *in-planta* in order to gain insights into plant stress (e.g., emerging from salt treatment or others) but also the provision of appropriate day-night cycles before any visible impact on growth appears. In our next experiments, we monitored K^+^/Na^+^ fluctuations in real-time in living plants under different stimuli, such as salt stress and light–dark rounds. On the one hand, Na^+^ transportation in plants is a key research area to study the salt impact on roadside plants (e.g., the effect of Na^+^-containing deicing agent, which is widely used in Stockholm) or monitor salt-tolerant plants for salt-land remediation.^25^ On the other hand, light–dark cycles can induce changes in ion fluxes and concentrations in some vegetal tissues.^29^ Light quality, intensity, and duration are known to affect both K^+^ uptake and its accumulation in plants.

**Figure 6:**
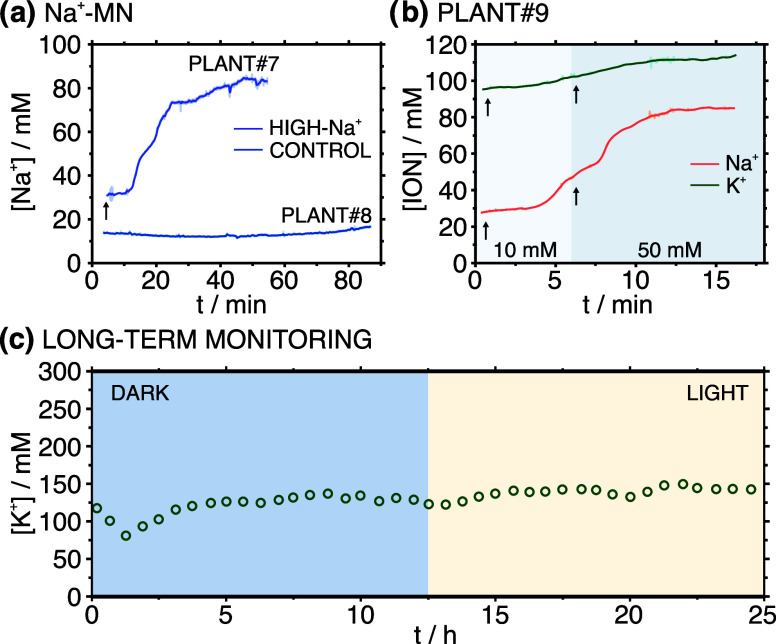
(a) Dynamic monitoring of Na^+^ concentrations in plant#7 and plant#8 using the developed Na^+^-MN sensor individual patch. (b) Simultaneous monitoring of K^+^ and Na^+^ concentrations in plant#9 after adding 10 and 50 mM NaCl into the culture solution. The developed K^+^/Na^+^-MN dual sensor patch was used. (c) Monitoring of the K^+^ concentration with the developed K-MN sensor individual patch along a dark-light cycle experienced by plant#10.

Starting with the salt-stress investigation, plant #7 and plant #8 were cultivated in the hydroponic solution (i.e., commercial culture solution without using soil) for 24 h. Then, the Na^+^-MN sensor patch was carefully mounted in the middle of the plants’ stems. Once the potential signal of the MNs was stabilized (±0.05 mV/min), NaCl was added into the hydroponic solution of plant #7 to obtain the final concentration of 200 mM, simulating salt-stress conditions. Plant #8 was used as a control, and hence, no NaCl was added to the culture solution. The results are provided in Figure 6a. As observed, an increase in the Na^+^ concentration appeared ca. 10 min after the addition of the NaCl (indicated by an arrow in the figure). In contrast, no significant changes in Na^+^ concentrations were observed in the control plant.

Our findings agree with previous studies that reported Na^+^ uptake from saline solutions accumulating in plant stems and leaves, though the authors measured Na^+^ content based on the basil’s total dry weight.^30^ There is strong evidence that salinity hinders normal plant growth, resulting in decreasing stem diameters, leaf fresh weight, and levels of many essential compounds. Advantageously, the developed MN patch offers Na^+^ monitoring with high spatiotemporal resolution. It evidenced that the Na^+^ concentration increase was indeed relatively fast: it took 20 min to reach a plateau of ca. 80 mM from the NaCl addition to the culture solution. Moreover, the Na^+^ dynamics could be monitored more deeply via several patches along the basil stem. Unlike traditional methods, the MN patch can monitor dynamic concentration profiles within confined stem areas, capturing changes as frequently as every second. Accordingly, concentration mappings could be achieved in the near future.

The K^+^/Na^+^-MN dual sensor patch was employed for monitoring K^+^ and Na^+^ levels simultaneously in plant #9. Na^+^ concentration in the culture solution was sequentially increased by adding NaCl to final concentrations of 10 and 50 mM. Figure 6b depicts the dynamic concentration profiles observed for the two ions. Addition timings for NaCl are represented by arrows in the figure. Attending to the Na^+^ profile, for a 10 mM NaCl concentration in the solution, the Na^+^-MN sensor detected a rise of Na^+^ in the stem within five minutes. This increase was somehow accelerated in the presence of 50 mM NaCl in the culture solution. Within five min, a clear and substantial increase was again observed, finally reaching a plateau corresponding to a ca. 82 mM Na^+^ concentration. Simultaneously with the Na^+^ increase, the K^+^-MN sensor revealed a slight increase in the K^+^ concentration. Normally, due to osmotic pressure, a higher Na^+^ level is expected to decrease the K^+^ uptake by the plant. However, there are some other complex mechanisms that have suggested the opposite, i.e., K^+^ ion also increases with increasing Na^+^, which is suggested by our outcomes.^31^

Additionally, the K^+^-MN sensor patch was employed to monitor K^+^ concentration dynamics in the stem for 24 h, considering a dark-light cycle. The results are shown in Figure 6c, considering an acquisition time of 30 min. According to the observed profile, the light–dark cycle did not exert a significant influence on the K^+^ concentration in the stem of plant #10. However, the light quality, intensity, and duration appear to play roles in regulating K^+^ levels. Based on this, a further experiment to be able to see drastic changes in K^+^ levels would involve the monitoring of subsequent day-night cycles under prolonged dark conditions, which is to happen in some countries like Sweden. Notably, we did not perform such an attempt to preserve the plant’s integrity.

**Figure 7:**
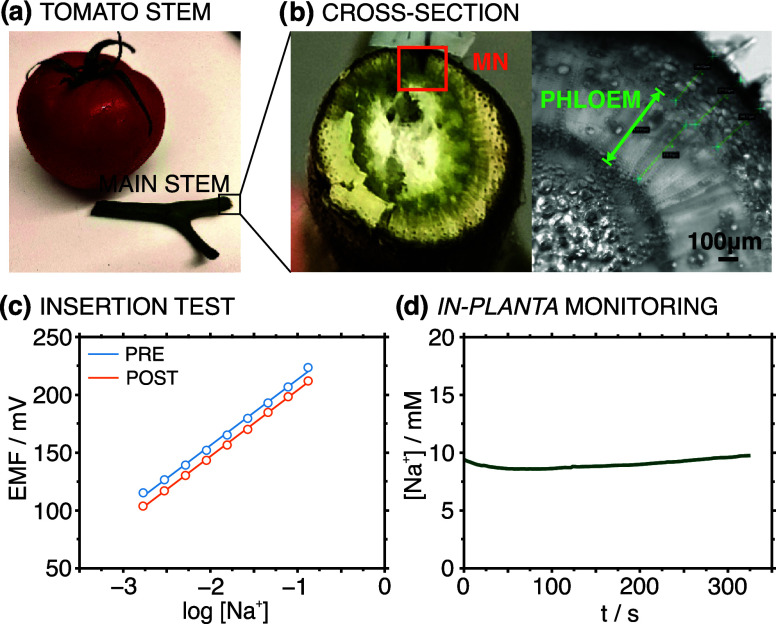
(a) Picture of the excised tomato’s main stem. (b) Physical (left) and microscopic (right) images of the cross-section of the main stem. (c) Calibration curves of the Na^+^-MN individual patch before and after the insertion in a tomato main stem. (d) Dynamic Na^+^ concentration monitored in tomato’s main stem.

Finally, the versatility of the developed MN sensor patch was demonstrated by monitoring Na^+^ levels in the main stem of a ripe tomato (Figure 7a). Although young tomato stems resemble those of basil, mature stems normally have a diameter of around 0.5–1 cm, i.e., much bigger than in basils (0.1–0.3 cm). Based on the MN’s length and stem layer, the MN is expected to monitor only sap in phloem, without reaching the xylem, as illustrated in Figure 7b. As the tomato stem is thicker and tougher to be punched, a slight change in the slope (0.32%) and intercept (2.42%) of the Na^+^ calibration graph was observed before and after stem insertion (Figure 7c). Figure 7d shows Na^+^ concentrations measured in an excised tomato stem using the Na^+^-MN sensor for 5 min. It was found that the Na^+^ level in the tomato’s stem was lower than that of basil (approximately 10 mM versus 23 mM).

Overall, the MN patch displayed adequate versatility to be used in different plants. Not only are indoor measurements achievable but also outdoor observations can be addressed by adding a temperature sensor to the patch to correct the slope in the calibration graph (this need is well-known for potentiometric ISEs). Moreover, the patch is suitable for both continuous and discrete measurements. Continuous observations will be relevant in cases of relatively fast reactions to stress factors (e.g., salinity changes), whereas the long-term natural development (e.g., growth) or adaptation of the plant to chronic stress may require weeks or months of daily discrete concentration values.

## Conclusions

This study shows the development of a dual potentiometric K^+^/Na^+^-MN sensor for the real-time monitoring of ion concentration profiles in plants. The MN sensor offers a rapid and accurate response, being in good agreement with gold standard methods when performing *in-planta* measurements. Thus, several cases of use are herein demonstrated. First, the usefulness of the K^+^/Na^+^-MN to monitor ion fluctuations under different stimuli, such as salt-stress and dark/light cycles, was shown in basil plants. Then, the versatility of the MN patch for detecting K^+^/Na^+^ was proved by monitoring other plants’ species, e.g., tomato plants. Overall, the developed MN patch has revealed significant potential for continuous monitoring of ions that are expected to fluctuate over time in (stressed) plants when laced in various organs and locations. The sensing platform represents a significant step forward from today’s state-of-the-art due to its high spatiotemporal resolution, which opens the door to a wide range of future applications and exciting research directions.

## Supporting Information Available

Experimental details; analytical performances; plant health status; microscopic images; reversibility and stability; and physical images of wounds (PDF)
