# Supplementary material for: Unveiling Potassium and Sodium Ion Dynamics in Living Plants with an In-Planta Potentiometric Microneedle Sensor
Source: ACS Sens. 2024 Sep 18;9(10):5214–23. doi: 10.1021/acssensors.4c01352 (PMC11519921; doi:10.1021/acssensors.4c01352)
Supplement: Supplementary file 1 — se4c01352_si_001.pdf [file se4c01352_si_001.pdf]

Supporting information for:

Unveiling Potassium and Sodium Ions Dynamics in Living Plants with an *In-Planta* Potentiometric Microneedle Sensor

Qianyu Wang[1], Águeda Molinero-Fernández[2], José-Ramón Acosta-Motos[3,4], Gastón A. Crespo[1,2,*], María Cuartero[1,2,*]

[1]Department of Chemistry, KTH Royal Institute of Technology, Teknikringen 30, SE-114 28, Stockholm, Sweden.
[2]UCAM-SENS, Universidad Católica San Antonio de Murcia, UCAM HiTech, Avda. Andres Hernandez Ros 1, 30107, Murcia, Spain.
[3]Plant Biotechnology for Food and Agriculture Group (BioVegA), Universidad Católica San Antonio de Murcia (UCAM), 30107, Murcia, Spain.
[4]Plant Biotechnology, Agriculture and Climate Resilience Group, Associate Unit of R&D+i CSIC-UCAM, 30100, Murcia, Spain.

(*) Corresponding Authors: Maria Cuartero (mariacb@kth.se). Gaston Crespo (gacp@kth.se)

Table of Contents

1. Experimental Section......3

Reagents, materials, and instrumentation. 3

Membrane compositions......3

2. Tables......4

Table S1. 4

Table S2. 4

Table S3. 4

Table S4. 5

Table S5. 5

Table S6. 6

Table S7. 6

Table S8. 6

Table S9. 6

3. Figures......7

Figure S1. 7

Figure S2. 7

Figure S3. 8

Figure S4. 8

Figure S5. 8

Figure S6. 9

Figure S7. 9

Figure S8. 10

Figure S9. 10

Figure S10. 11

Figure S11. 11

4. References......12

1. Experimental Section

Reagents, materials, and instrumentation. Analytical grade chloride salts of potassium (CAS-7447-40-7), sodium (CAS-7647-14-5), calcium (CAS-22691-02-7), ammonium (CAS-12125-02-9), magnesium (CAS-7786-30-3), bis(2-ethylhexyl) sebacate (DOS, ≥97% purity, CAS-122-62-3), polyurethane (PU, CAS-51-79-6), polyvinyl butyral (PVB, CAS-63148-65-2), tetrahydrofuran (THF, CAS109-99-9), sodium tetrakis[3,5- bis(trifluoromethyl)phenyl]borate (NaTFPB, > 98%, CAS-79060-88-1), sodium ionophore X (selectophore grade, CAS-97600-39-0), potassium ionophore I (valinomycin, selectophore grade, CAS-2001-95-8), potassium tetrakis (4-chlorophenyl)borate (KTClPB, > 98%, CAS-14680-77-4), thionyl chloride ($SOCl_2$, ≥97% purity, CAS-7719-09-7), octadecylamine (ODA, >99%, CAS-124-30-1), dimethylformamide (DMF, >99%, CAS-68-12-2), and multi-walled carbon nanotubes (MWCNTs, CAS-308068-56-6) were purchased from Sigma-Aldrich. Absolute ethanol (CAS-64-17-5) and methanol (CAS-67-56-1) were purchased from VWR.

The Parafilm used as the support substrate was purchased from VWR (291-0057). The stainless-steel microneedles were obtained by cutting them from a commercial medical grade microneedle roller (Dermaroller, Local Supplier, Sweden). Silver/silver chloride (C2131007D3) and carbon (C2030519P4) pastes were purchased from Sun Chemical. The glue used for fixing the microneedles from the upper part of the substrate was a strong, quick-drying and water-resistant type, equipped with a pointed nozzle for high precision dripping, suitable for bonding plastics and metals (Loctite, Henkel Norden AB).

Electromotive force (EMF) was measured by an EMF16 multichannel data acquisition device (Precision Electrochemistry EMF Interface, Lawson Labs, Inc.) with a high input impedance (10^13 ohms typical) and 4 Hz noise filter. A commercial double junction Ag. AgCl / 3M KCl reference electrode (6.0726.100, Metrohm AB, Sweden) was used in some steps of the in vitro evaluation of the microneedles. Microscopic images were taken with an inverted optical microscope (Nikon Eclipse Ti2, Japan). Plant sap ion concentrations were measured using ion chromatography instrument with a conductivity detector (850 Professional IC, Metrohm AB, Sweden). Separation column of model Metrosep C6-150/4.0 (6.1051.420, Metrohm AB, Sweden) and 919 IC Autosampler Plus (2.919.0020) were used. Ultrasonic dissolving was performed using an ultrasonic bath (USC200TH 142-6047, VWR). Liquid samples were mixed using a vortex mixer (10153-810, VWR). All solutions were prepared using double-deionized water of 18.2 $M \cdot cm^{-1}$ from the Milli-Q water systems (Merck Millipore).

The dual MN sensor was integrated with a portable and wireless multi-potentiometer board previously reported by our group.[1,4] To verify the accuracy of the device, the sensing performance was evaluated in comparison with the benchtop potentiometric workstation. Both (i.e. portable and benchtop potentiometers) revealed very similar slopes: 58.1 and 58.5 mV dec^{-1} and intercepts of 237.0 and 288.2 mV for the K^+ calibration in artificial sap. Accordingly, we confirmed that the MN patch herein developed can be used with the portable board.

Membrane compositions. For the potassium-selective membrane, 2.2 mg of the potassium ionophore I, 0.5 mg KTClPB, 33 mg PU, and 66 mg DOS were dissolved in 1 mL of THF. For the sodium-selective membrane, 0.1 mg of the sodium ionophore X, 0.49 mg NaTFPB, 33 mg PU, and 66 mg DOS were dissolved in 1 mL of THF.[1] For the reference membrane, 78 mg of PVB and 50 mg NaCl were dissolved in 1 mL of methanol.[2] The membrane solutions were mixed using the vortex mixer. Functionalized multiwalled carbon nanotubes (f-MWCNTs) were prepared as reported elsewhere.[3] Fine powders of f-MWCNTs were dissolved in absolute ethanol reaching a concentration of 1 mg/mL, with the solution being mixed in the ultrasonic bath.

2. Tables

Table S1. Calibration parameters of the Na^+-MN based on different substrates as well as without and with an external PU layer.

Outer Membrane	Substrate	Slope (mV dec^{-1})	Intercept (mV)	Number of MNs tested
–	Silicone rubber	54.7 ± 1.0	273.9 ± 20.0	7
–	Parafilm	57.2 ± 2.1	260.0 ± 22.9	6
PU	Parafilm	57.4 ± 1.8	304.7 ± 27.0	7

Table S2. Calibration parameters before and after several insertions into the stem. Na^+-MNs were prepared without and with the external PU layer.

	Insertions	Slope (mV dec^{-1})	% of variation, slope	Intercept (mV)	% of variation, intercept
Without PU	Before	54.3	–	272.8	–
	1	53.9	0.5	251.2	5.8
	5	53.2	1.4	232.7	11.2
With PU	Before	56.8	–	308.4	–
	1	56.8	0	309.6	0.3
	5	57.2	0.5	309.7	0.3

Table S3. Calibration parameters and response time of 6 patches based on Na^+- and RE-MNs. Additionally, the RE-MN was substitute by a commercial Ag/AgCl reference electrode (RE-COM) to confirm the appropriate behavior of the RE-MNs. The response time was calculated as t_{95} within the linear range of response.

<table>
<tr><th rowspan="2">Patch No.</th><th colspan="2">Slope (mV dec^{-1})</th><th colspan="2">Intercept (mV)</th><th colspan="2">LRR (M)</th><th colspan="2">LOD (M)</th><th colspan="2">Response time, t_{95} (s)</th></tr>
<tr><th>MN</th><th>COM</th><th>MN</th><th>COM</th><th>MN</th><th>COM</th><th>MN</th><th>COM</th><th>MN</th><th>COM</th></tr>
<tr><td># 1</td><td>58.2</td><td>58.3</td><td>345.4</td><td>341.6</td><td rowspan="6">10^{-5}–$10^{-0.75}$</td><td rowspan="6">10^{-5}–$10^{-0.75}$</td><td rowspan="6">8.8×10^{-6}</td><td rowspan="6">7.9×10^{-6}</td><td rowspan="6">3.0–5.0</td><td rowspan="6">3.0–5.0</td></tr>
<tr><td># 2</td><td>54.8</td><td>53.3</td><td>348.2</td><td>345.2</td></tr>
<tr><td># 3</td><td>56.4</td><td>55.7</td><td>347.8</td><td>349.4</td></tr>
<tr><td># 4</td><td>57.5</td><td>56.8</td><td>346.5</td><td>360.4</td></tr>
<tr><td># 5</td><td>55.6</td><td>55.5</td><td>357.9</td><td>380.9</td></tr>
<tr><td># 6</td><td>58.6</td><td>56.8</td><td>338.5</td><td>353.3</td></tr>
</table>

Table S4. Logarithmic selectivity coefficients ($logK_{I,J}^{pot}$) for the main ions present in sap. I = main ion. J = interfering ion.

Sensor patch	Main ion (I)	Interfering ion (J)	$logK_{I,J}^{pot}$	Expected concentration of J in sap (mM)	Needed value
Na^+-MN	Na^+	K^+	–2.6 ± 0.02	50–150	–2.1
	Na^+	Ca^{2+}	–4.0 ± 0.05	20	–2.2
K^+-MN	K^+	Na^+	–2.7 ± 0.04	20–30	–1.7
	K^+	Ca^{2+}	–3.1 ± 0.05	20	–2.1

Table S5. Calibration parameters of individual K^+/Na^+-MN sensor patches in artificial sap and ultrapure water in the concentration range from 10^{-3} to $10^{-0.75}$ M. Three consecutive calibrations were performed in each case.

**K^+-MN patch**			
**Matrix**	**Testing sequence**	**Slope (mV dec^{-1})**	**Intercept (mV)**
Artificial sap	1st	57.7	307.2
	2nd	58.9	313.6
	3rd	58.9	316.3
Ultrapure water	4th	56.9	310.4
	5th	57.7	312.9
	6th	57.2	317.0
**Na^+-MN patch**			
**Matrix**	**Testing sequence**	**Slope (mV dec^{-1})**	**Intercept (mV)**
Artificial sap	1st	53.7	322.6
	2nd	53.7	323.2
	3rd	53.0	321.6
Ultrapure water	4th	56.7	225.2
	5th	56.7	326.2
	6th	55.9	324.2

Table S6. Calibration parameters of individual K^+- and Na^+-MN sensors in ultrapure water and artificial sap (a-sap) in an extended concentration range $10^{-6.5}$ to $10^{-0.75}$ M.

	Na^+-MN		K^+-MN	
	**Water**	**a-sap**	**Water**	**a-sap**
Slope (mV dec^{-1})	56.3±0.09	52.2±0.05	55.3±0.03	53.2±0.02
Intercept (mV)	296.4±1.3	292.0±0.03	319.1±4.8	318.0±2.8
LRR (M)	10^{-5}–$10^{-0.75}$	$10^{-3.5}$–$10^{-0.75}$	10^{-4}–$10^{-0.75}$	10^{-3}–$10^{-0.75}$
LOD (M)	7.5×10^{-6}	2.2×10^{-4}	2.9×10^{-5}	2.8×10^{-4}
Response time, t_{95} (s)	6.06×10^{-5}	5.9×10^{-4}	3.0–5.0	3.0–5.0

Table S7. Recovery tests in sap samples using individual K^+- and Na^+-MN-based patches.

Sap No.	Sensor patch	Fortified (mM)	Detected (mM)	Recovery rate
# 1	Na^+-MN	1	1.02	102.2%
# 2	Na^+-MN	6	6.19	103.2%
# 3	K^+-MN	1	1.10	110.1%
# 4	K^+-MN	50	51.56	103.1%

Table S8. Comparison of two K^+-MN sensors detecting the K^+ concentration in the sap.

Sap No.	Test group No. 1	Measured K^+ (mM)	Test group No. 2	Measured K^+ (mM)	Difference
# 1	K^+-MN #1	108.8 ± 0.2	K^+-MN #2	118.8 ± 0.2	8.4%
# 2	K^+-MN #3	108.7 ± 1.8	K^+-MN #4	95.5 ± 0.04	13.8%
# 3	K^+-MN #5	99.1 ± 1.4	K^+-MN #6	97.2 ± 0.3	1.9%
# 4	K^+-MN #7	103.2 ± 0.9	K^+-MN #8	99.02 ± 0.2	4.2%
# 5	K^+-MN #9	95.01 ± 0.5	K^+-MN #10	96.05 ± 0.2	1.1%
# 6	K^+-MN #11	121.2 ± 0.9	K^+-MN #12	122.7 ± 0.2	1.2%

Table S9. Health status of basil plants registered for *in-planta* testing.

Plant No.	Overall height (cm)	Number of leaves	Color of leaves	Stem diameter (mm)	# new sprouts*	Treatment
# 1	18	8	Vibrant green, yellowing in one leaf	2.5	5	Cultivate in 30 mM NaCl for 24 h
# 2	12	5	Vibrant green	1.1	4	/
# 3	22	11	Vibrant green	2.4	9	/
# 4	17	6	Vibrant green, yellowing in one leaf	2	6	/
# 5	15	8	Vibrant green	2.2	4	/
# 6	16	8	Vibrant green	2	5	/

*Newly grown sprouts from the plant's stem.

3. Figures

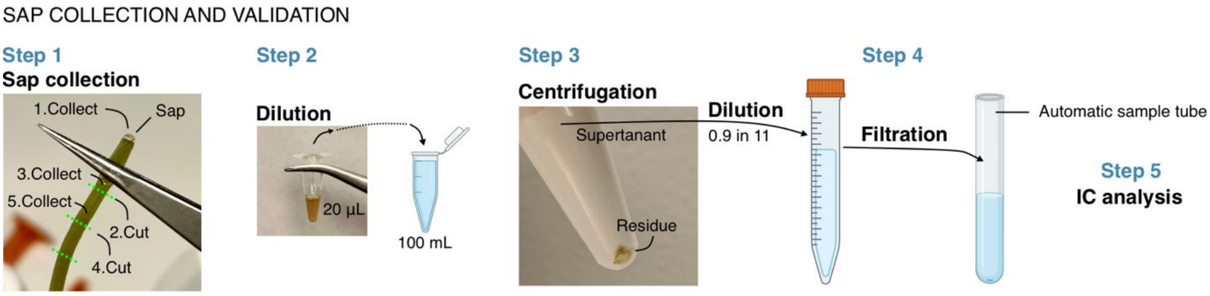

Figure S1. Sap collection and IC validation procedure.

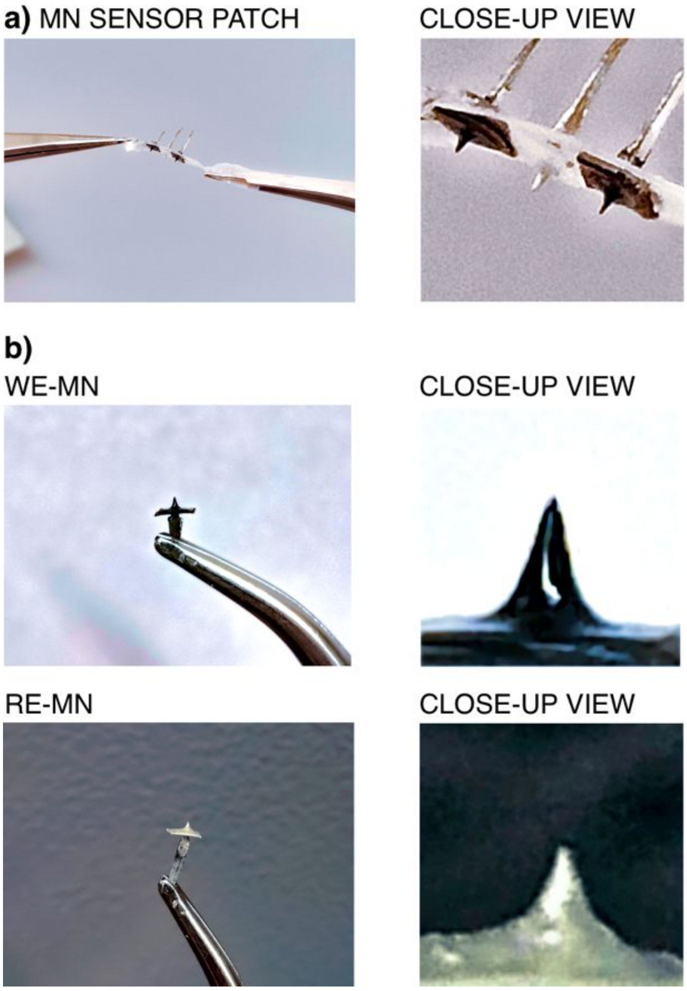

Figure S2. Real photos of (**a**) the MN sensor patch and its close-up view and (**b**) individual WE/RE-MNs and their close-up views.

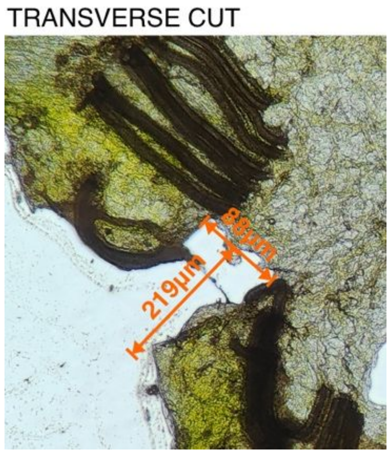

Figure S3. Transverse cut of the basil stem after being inserted with a MN sensor.

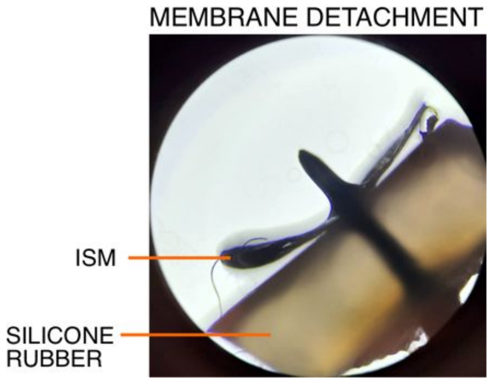

Figure S4. Membrane detachment from the silicone rubber substrate of the ion-selective MN sensor patch during *in vitro* testing.

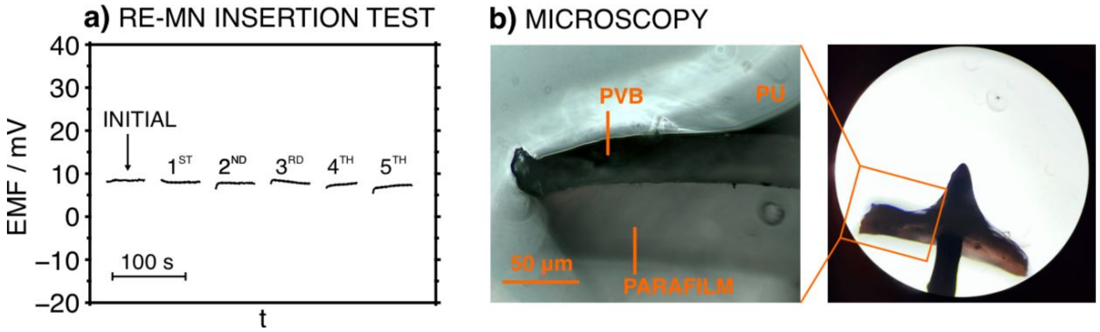

Figure S5. (**a**) Dynamic potentiometric response of the MN-RE in 0.1 M KCl solution before and after one and five insertions into the stem. (**b**) Microscopic photo of the MN-RE after being inserted into a basil plant after 5 times. Magnification of the parafilm-PVB-PU interfaces, discharging any detachment.

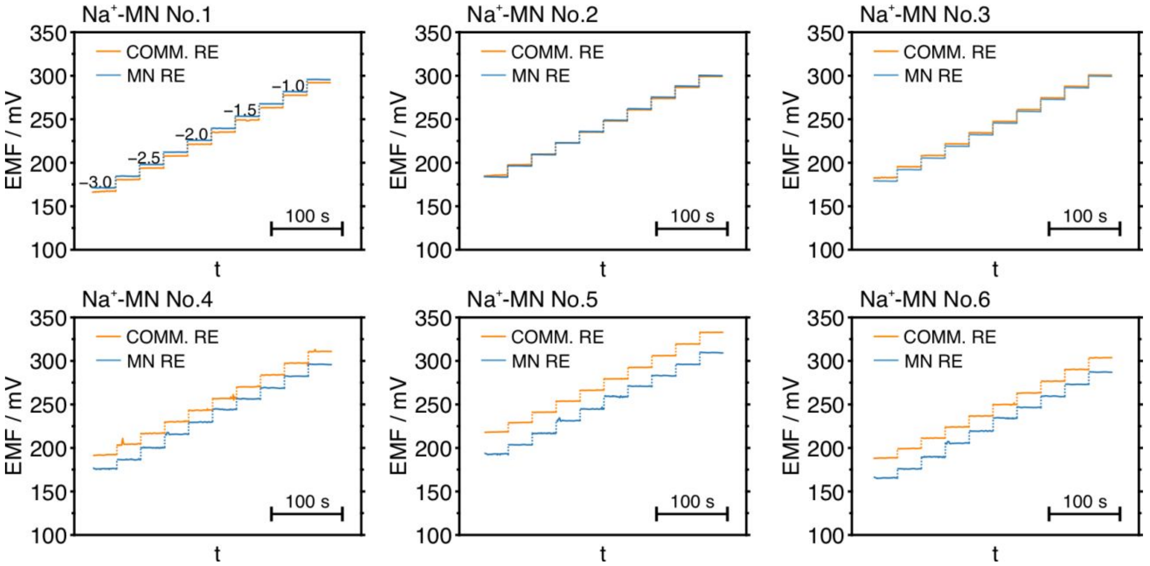

Figure S6. Dynamic responses of six analogous patches (Na^+- and RE-MN). The results with the alternative use of a commercial Ag/AgCl reference electrode (RE-COM) are also provided. Background: ultrapure water.

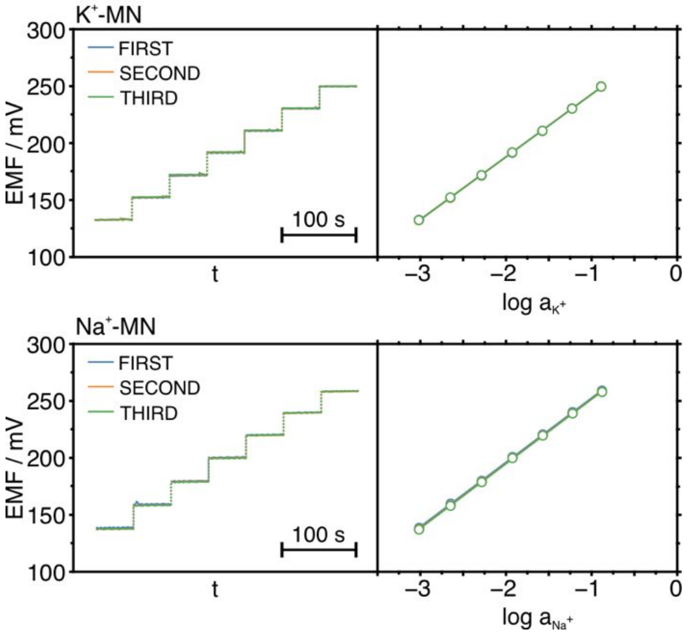

Figure S7. Dynamic responses and the corresponding calibrations for individual K^+ (top) and Na^+ (bottom) patches performing three consecutive experiments.

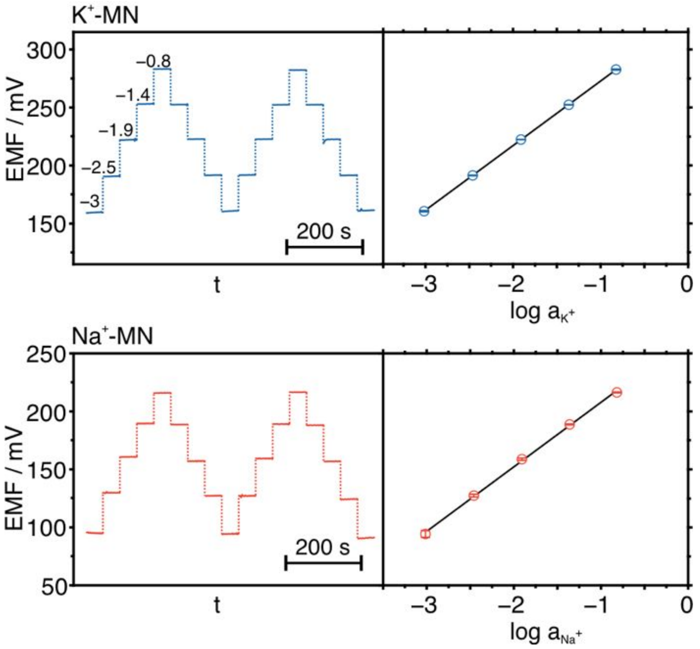

Figure S8. Dynamic responses and the corresponding calibrations obtained in the reversibility test of individual K^+ (top) and Na^+ (bottom) MN patches.

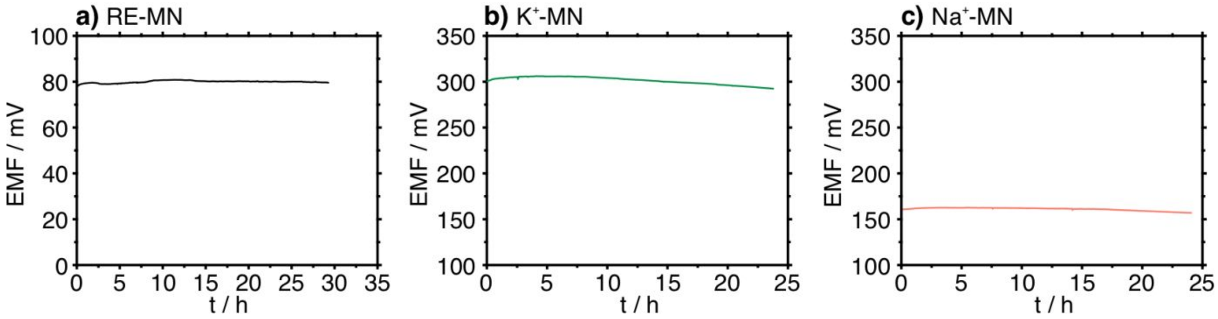

Figure S9. Long-term stability of (**a**) RE-MN, (**b**) K^+-MN, (**c**) Na^+-MN in artificial sap.

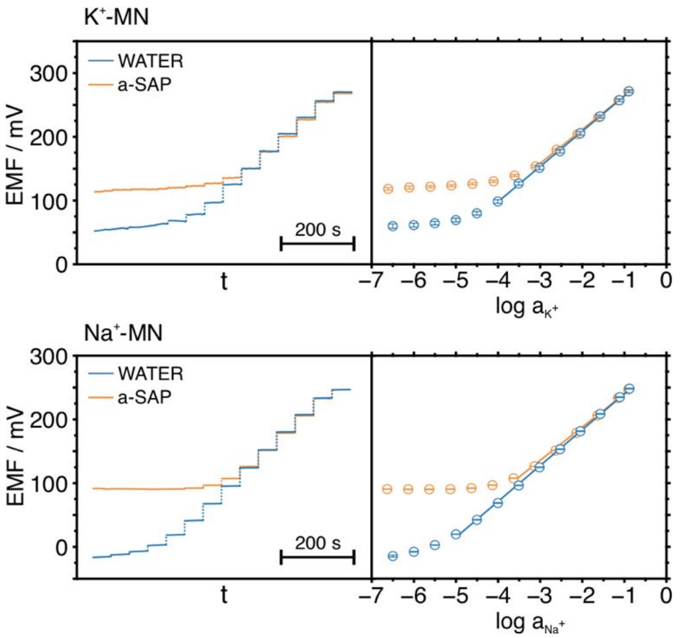

Figure S10. (Top) Dynamic responses and the corresponding calibrations of the K^+-MN individual patch in artificial sap and ultrapure water in the K^+ range of response of $10^{-6.5}$–$10^{-0.75}$ M. (Bottom) Dynamic responses and the corresponding calibrations of the Na^+-MN patch in artificial sap and ultrapure water in the Na^+ range of response of $10^{-6.5}$–$10^{-0.75}$ M. Error bars stand for three consecutive measurements.

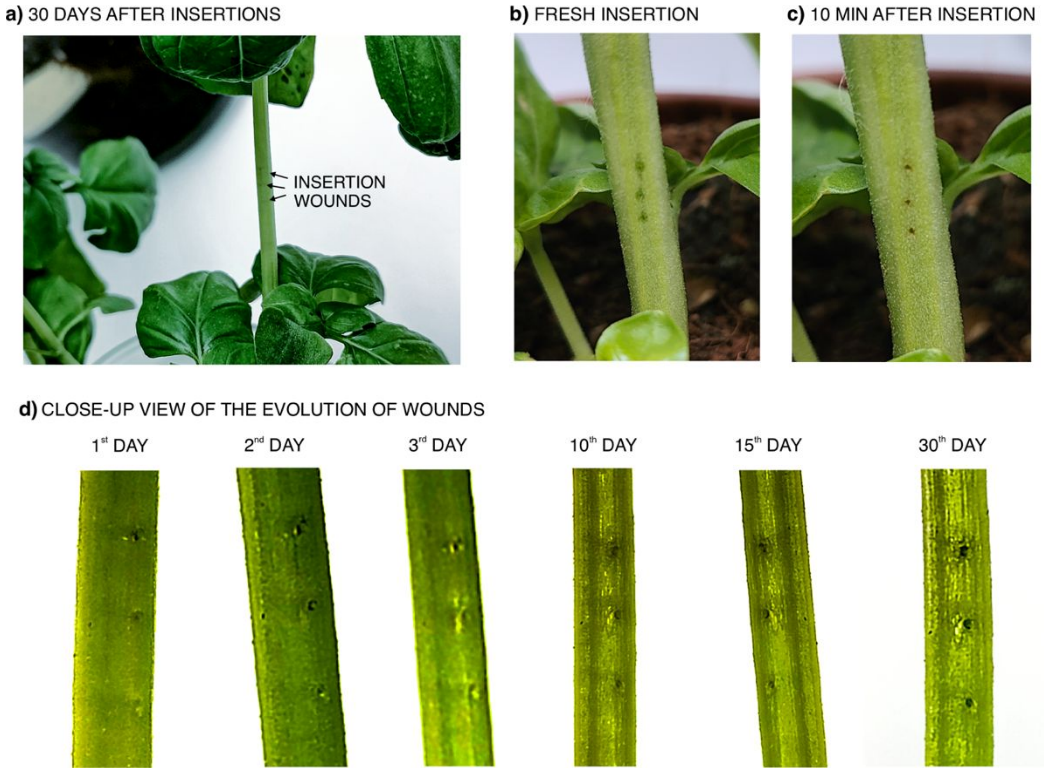

Figure S11. (**a**) Picture of the basil plant 30 days after the MN patch insertion on the stem. Close-up view of the insertion holes appearing (**b**) immediately after the insertion and (**c**) 10 minutes after the insertion. (**d**) Close-up view of the evolution of the wounds with time.

4. References

(1) Molinero-Fernández, Á.; Casanova, A.; Wang, Q.; Cuartero, M.; Crespo, G. A. In Vivo Transdermal Multi-Ion Monitoring with a Potentiometric Microneedle-Based Sensor Patch. *ACS Sens.* **2022**, *8* (1), 158-166.
(2) Guinovart, T.; Crespo, G. A.; Rius, F. X.; Andrade, F. J. A reference electrode based on polyvinyl butyral (PVB) polymer for decentralized chemical measurements. *Anal. Chim. Acta* **2014**, *821*, 72-80.
(3) Yuan, D.; Anthis, A. H.; Ghahraman Afshar, M.; Pankratova, N.; Cuartero, M.; Crespo, G. A.; Bakker, E. All-solid-state potentiometric sensors with a multiwalled carbon nanotube inner transducing layer for anion detection in environmental samples. *Anal. Chem.* **2015**, *87* (17), 8640-8645.
(4) Molinero-Fernandez, Á.; Wang, Q.; Xuan, X.; Konradsson-Geuken, Å.; Crespo, G. A.; Cuartero, M. Demonstrating the Analytical Potential of a Wearable Microneedle-Based Device for Intradermal CO2 Detection. *ACS Sens.* **2024**.
(5) Meier, P. C. Two-parameter Debye-Hückel approximation for the evaluation of mean activity coefficients of 109 electrolytes. *Anal. Chim. Acta* **1982**, *136*, 363-368.
(6) Bakker, E.; Pretsch, E.; Bühlmann, P. Selectivity of potentiometric ion sensors. *Anal. Chem.* **2000**, *72* (6), 1127-1133.
